# Development and validation of an antimicrobial stewardship clinical decision-support tool to improve the management of urinary tract infections versus asymptomatic bacteriuria in hospitalized patients

**DOI:** 10.1017/ash.2024.433

**Published:** 2024-11-19

**Authors:** Mayan Gilboa, Renata Boatwright, Veronica Salazar, Julio C. Simon, Brianna North, Christine Vu, Ana Vega, Kailynn Jennings Deronde, Rossana Rosa, Lilian M Abbo

**Affiliations:** 1 Faculty of Medicine, Tel Aviv University, Tel Aviv-Yafo, Israel; 2 Department of Infection prevention and control, Jackson Health System, Miami, FL, USA; 3 Infection Prevention and Control Unit, Sheba Medical Center, Ramat Gan, Israel; 4 Department of Pharmacy, Jackson Health System, Miami, FL, USA; 5 Department of Medicine, Division of Infectious Diseases, University of Miami Miller School of Medicine, Miami, FL, USA

## Abstract

**Objective:**

To assess the effectiveness of a 3-question decision-support tool to guide the diagnosis and treatment of urinary tract infections (UTIs) in acute care hospitalized patients as an antibiotic and diagnostic stewardship initiative.

**Design:**

Retrospective cohort study.

**Setting:**

Four acute care hospitals within the same health system in Miami, FL.

**Patients:**

124, admitted from the emergency department and hospitalized adult patients, treated with antibiotics for the indication of a UTI between March and April 2023.

**Intervention:**

We developed a 3-step clinical decision-support tool (CDST) to evaluate the appropriateness of urine cultures and antibiotic treatment. The tool’s recommendations when deciding to prescribe antibiotics were compared with the actual need for treatment throughout the hospitalization, up to the time of patient discharge.

**Results:**

Overall, 31% of antibiotics prescribed for UTIs were inappropriate and met the criteria for asymptomatic bacteriuria (ASB) based on the CDST. Prospective implementation of the decision-support tool could potentially reduce antibiotic use by 33.6%, corresponding to 265 days of unnecessary therapy. The sensitivity and specificity of the tool were calculated to be 98.6% and 100%, respectively, indicating high accuracy in identifying the need for antibiotic treatment. Urinalysis alone was insufficient to differentiate between symptomatic UTIs and ASB, with leukocyturia present in 95.3% of UTI cases and 94.6% of ASB cases (*P* = 0.87).

**Conclusions:**

Implementing a 3-question CDST may reduce unnecessary laboratory work-up and treatment for ASB improving the diagnostic and antimicrobial stewardship of UTIs.

## Introduction

Urinary tract infections (UTIs) are one of the leading bacterial infections both in community and hospital settings and globally affect more than 400 million individuals annually.^1,2^ UTIs account for 18.6% of all antibiotic prescriptions in hospitals,^3^ emphasizing the need for antimicrobial stewardship management strategies. The economic impact of UTI-related hospitalizations is notable, with annual costs reaching approximately $2.8 billion.^4^

The diagnosis of UTIs can be challenging, as it relies on clinical symptoms. Positive urinalysis or urine cultures on their own cannot confirm UTI diagnosis, as the presence of bacteria in the urine without the presence of symptoms can be asymptomatic bacteriuria (ASB) and most times does not warrant treatment; antibiotics for ASB are indicated in specific cases such as pregnancy before an endourological procedure or a recent renal transplantation.^5^ The distinction between a true UTI and ASB may pose difficulties, especially among populations such as the elderly or those with altered mental status who are unable to communicate their symptoms effectively. Although the incidence of UTIs increases with age—for example, women over the age of 85 have a 30% annual UTI incidence rate^6^ and so does the prevalence of ASB. As a consequence, the risk of receiving unnecessary antibiotics for the treatment of ASB is elevated in patients who are elderly or have dementia.^7,8^

The consequences of UTI overdiagnosis and overtreatment include extended hospital stays and a greater risk of *Clostridioides difficile* infection.^7,9^ Furthermore, the treatment of ASBs has led to increasing antimicrobial resistance, stressing the need for judicious antibiotic use.^10^ Despite this knowledge, a large portion of patients undergo unnecessary laboratory testing for UTIs without displaying symptoms, and the results of an abnormal test should not dictate treatment alone.^11–13^

Healthcare providers’ approaches to ordering unnecessary urine cultures and prescribing unnecessary antibiotics are influenced by a mix of factors, including psychological aspects such as anxiety and overcautiousness, alongside external pressures like institutional norms and patient expectations.^14^ Nonetheless, there are also significant knowledge gaps regarding this topic among many providers, with studies showing that only a minority of resident physicians can accurately identify the difference between ASB and UTI.^15^ In tackling these difficulties, the creation of decision-support tools to reduce unnecessary urine culture testing has shown promise.^16^ This study utilizes a 3-question clinical decision-support tool (CDST), derived from the Infectious Diseases Society of America (IDSA) guidelines.^5,17^ This antimicrobial stewardship tool was designed to improve the decision-making process before considering a UTI diagnosis by either obtaining urinary cultures or by prescribing antibiotics in newly hospitalized patients without indwelling urinary catheters; by using this tool, we aim to highlight some of the challenges associated with UTI overdiagnosis and overtreatment in acute care settings.

## Methods

This study was conducted as a retrospective cohort analysis within Jackson Health System, one of the largest public health systems in the United States, comprising over 2,100 beds across 4 different facilities throughout the Miami-Dade County area. Utilizing clinical surveillance software (Vigilanz Corp, Chicago, IL), we identified all cases with an antibiotic order for a UTI indication from March to April 2023. Exclusion criteria included patients younger than 18, patients discharged from the emergency department (ED) without hospitalization, and those on 24-hour observation or with onetime orders, resulting in a final cohort of 124 hospitalized patients. A comprehensive review of each case from admission to discharge was performed by both an infectious diseases physician and a clinical pharmacist specializing in antibiotic stewardship. Data collected included facility, line of service, patient age, sex, symptoms observed when the antibiotic was prescribed or culture obtained and throughout the hospitalization, and details of antibiotic treatments, such as type, dosage, and duration as well as further outpatient prescriptions.

UTI cases were classified into specific syndromes: ASB (absence of documented urinary symptoms, fever, or clinical instability), cystitis (presence of dysuria without fever), pyelonephritis (symptoms including flank pain, fever, or clinical instability without another etiology),^18^ and other infections requiring antibiotics. This classification allowed for a detailed analysis of UTI management practices. Laboratory parameters collected included urinalysis findings before antibiotic initiation.

**Figure 1.**
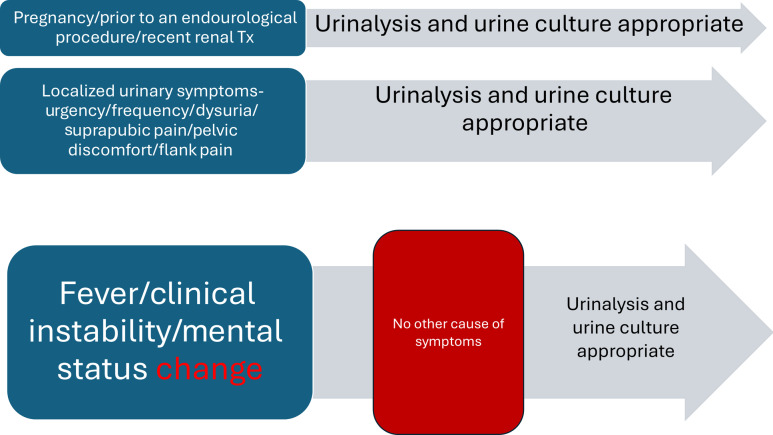
A 3-question support tool, Question 1: Is the patient pregnant? Is the request before an endourological procedure? Did the patient undergo a renal transplant in the past 60 days? If yes, the criteria for treatment of asymptomatic bacteriuria is fulfilled and a urinalysis and urine culture are appropriate regardless of symptoms. Question 2: Does the patient have localized urinary symptoms such as urgency, frequency, dysuria, suprapubic pain, pelvic discomfort, or flank pain? If yes, a urinary tract infection is highly suspected, and a urinalysis and urine culture are appropriate. Question 3: Does the patient have a fever, clinical instability, or an acute change in his mental status such as delirium? If yes, and if there is no alternate explanation for the symptoms, a urinalysis and a urine culture are appropriate. In this case, a UTI is a diagnosis of exclusion when no other etiology is present.

To evaluate the appropriateness of urine studies and antibiotic prescribing for UTIs, we utilized a novel CDST based on IDSA guidelines for managing ASB and infections in long-term care facilities.^5,17^ These guidelines were selected for their specific considerations in diagnosing UTIs in patients who are unable to provide a coherent history, addressing a common challenge identified during our case examinations. The decision-support framework involved the following process (Figure 1): (1) Screen for specific clinical scenarios that would justify the testing and treatment of ASB (eg, pregnancy, impending endourological procedures, renal transplantation within the last 60 days). Affirmative cases automatically warrant urine culture and antibiotic treatment if positive. (2) Assess for localized urinary symptoms such as urgency, frequency, dysuria, suprapubic pain, pelvic discomfort, or flank pain. The presence of any such symptoms deems urine studies and antibiotic treatment appropriate. (3) Evaluate for systemic symptoms and signs such as fever, clinical instability, or changes in mental status. If systemic signs are present without an alternate explanation, urine studies, and antibiotic treatment would be deemed appropriate.

In evaluating the effectiveness of the decision-support tool, sensitivity and specificity were calculated by comparing the tool’s recommendations for the testing and treatment of UTI with the information available at the time of prescribing against the true need for treatment decided by the case reviewer after a comprehensive inspection of the complete hospitalization in the Electronic Medical Redord (EMR). The tool’s performance was assessed by identifying cases that matched the criteria for requiring urine studies and antibiotic treatment for a urinary pathogen as confirmed by clinical and laboratory findings. Sensitivity was determined by the proportion of true positives correctly identified by the model, while specificity was calculated based on the proportion of true negatives accurately recognized.

Statistical analysis was conducted using R version 4.3.0, employing descriptive statistics, *t* tests, and chi-square tests focused on the cohort’s characteristics and outcomes. Missing data were excluded from the specific analyses affected.

This study was conducted as part of an institutional quality improvement initiative aimed at improving antimicrobial and diagnostic stewardship.

## Results

The study included 124 cases, with a median age of 64 years old (interquartile range [IQR]: 46–74.25). Among these, 75 (60%) were female, including 1 pregnant patient. Urine cultures were performed before urological procedures in 8 instances.

Based on predefined criteria, fifty patients (40%) were classified as pyelonephritis, and 15 (12%) as cystitis. Additionally, 13 (11%) did not have a UTI but were identified with other non-urinary sources of infection (Table 1). These included pneumonia (n = 4), intra-abdominal infections (n = 4), osteomyelitis and infected decubitus ulcers (n = 2), coronavirus disease 2019 (n = 1), cellulitis (n = 1), and epididymitis (n = 1). ASB was observed in 46 individuals (37%), of whom 8 (17%) were indicated for antibiotic treatment in anticipation of upcoming urological procedures. The 1 pregnant patient included in the cohort had symptomatic cystitis.

**Table 1.** Classification of UTI diagnoses—summary of symptoms among patients with asymptomatic bacteriuria, pyelonephritis, cystitis, and other infections requiring antibiotics

	Asymptomatic bacteriuria (46)	Cystitis (15)	Pyelonephritis (50)	Other infection (13)
Urgency/frequency/ dysuria	0	12	7	0
Pelvic discomfort/suprapubic pain/flank pain	1*	6	27	1
Fever	0	0	28	3
Rigors	0	2	9	1
Clinical instability	2**	0	16	3
Fall/syncope	10	1	2	0
Mental status change	9***	0	12	3
Cloudy/foul-smelling urine	2	1	4	0
Foley catheterization	2	4	2	3
Other urinary instrumentation (stent/nephrostomy)	2	4	2	3
Nephrolithiasis	1	1	18	1
Poor historian	12	4	12	8

* Patient after trauma with a kidney laceration.

** Patient with cardiac arrest and cardiogenic shock, patient after trauma with carotid artery laceration and hypovolemic shock.

*** Etiologies: neurological—seizure; psychiatric—psychotic event in a patient with a known diagnosis of schizophrenia; metabolic—severe hyponatremia, cirrhosis with hyperammonemia, alcohol intoxication, and hallucinations. Other reasons for hypoperfusion (cardiogenic and hypovolemic shock).

### Description of symptoms:

Symptom documentation from medical records revealed that 29% (36 of 124) were considered “poor historians,” primarily due to conditions such as advanced dementia, acute psychosis, intoxication, and medically unstable conditions that impeded their ability to communicate symptoms effectively. Specific symptoms associated with each diagnosed condition are outlined in Table 1. Overall, 38 patients (31%) were treated without documented evidence of urinary symptoms or a specific indication for treating ASB. There was 1 patient who experienced pelvic pain following a kidney trauma that resulted in a laceration. Additionally, 9 patients with ASB experienced altered mental status; however, these episodes could have been attributed to other causes, as outlined in Table 1

### Sensitivity and specificity of the decision-support tool

To evaluate the sensitivity and specificity of the decision-support tool, we compared the tool’s indication for a urine culture at the time it was obtained and for antibiotic treatment when initiated with the information available at that time, against the true need for urine studies and treatment, decided by the case reviewer after a comprehensive inspection of the complete course of the hospitalization in the EMR. Seventy-three cases were identified by the tool as requiring testing and treatment, and upon review of the entire hospital stay, antibiotic treatment for a urinary pathogen was indeed necessary in all instances. Of the 51 cases deemed not to require urine culture by the tool, only 1 eventually needed antibiotics for a UTI (a patient with an initial diagnosis of pneumonia who developed *E. coli* bacteremia and bacteriuria. The presence of this typical urinary pathogen in both the blood and urine suggests the pathogenesis of a UTI concurrent with pneumonia). Based on these outcomes, the sensitivity of the 3-question model was calculated to be 98.6%, with a specificity of 100%.

### Urinalysis results

We further investigated whether certain urinalysis markers could indicate a higher likelihood of an actual UTI rather than ASB. This involved comparing urinalysis results from 27 cases of ASB, where data were available, against those from 58 UTI cases. We found that 95.3% of UTI cases exhibited leukocyturia compared to 94.6% in ASB cases (*P* = 0.87), 59.4% of UTIs had detectable bacteria versus 68.4% in ASB cases (*P* = 0.66), and 21.9% of UTIs showed nitrates compared to 18.4% in the ASB group (*P* = 0.37). Although leukocyte counts were higher in samples from UTI cases (863.4 cells/µL) than in ASB cases (164.9 cells/µL), this difference did not reach statistical significance (*P* = 0.08).

### The burden of antibiotic treatment of patients with ASB

**Figure 2.**
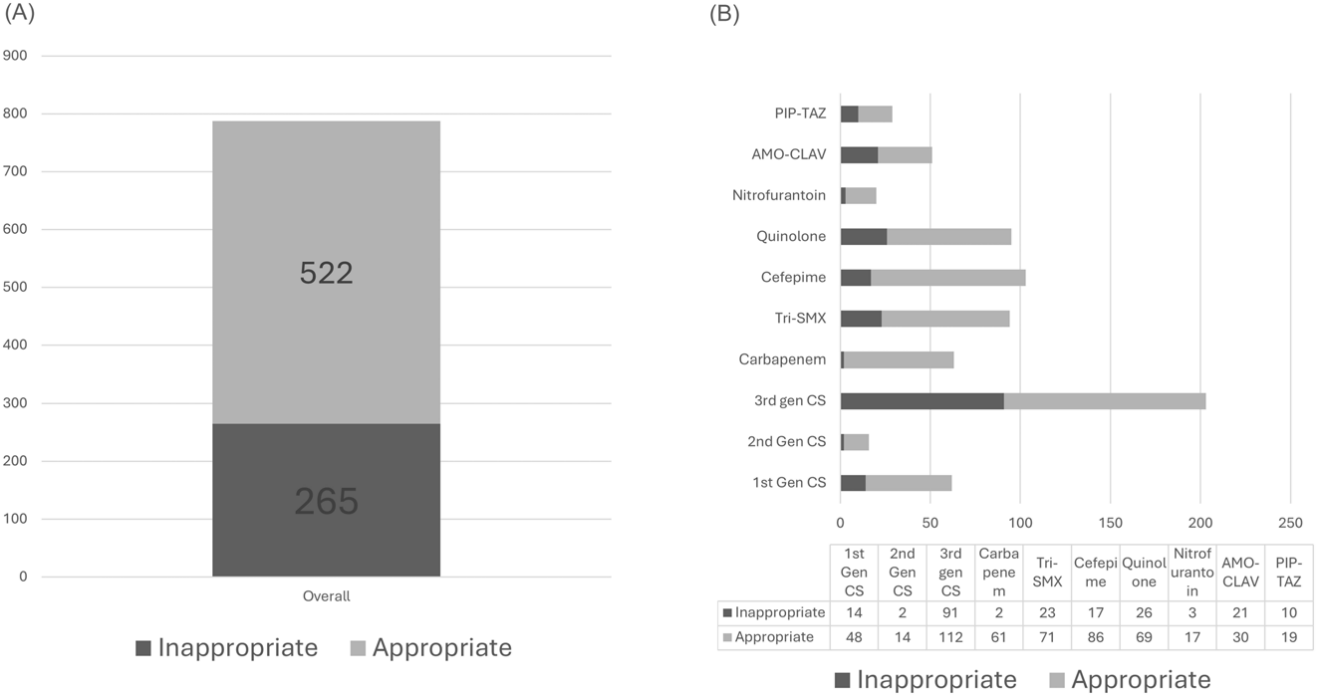
Antibiotic treatment by the appropriateness of urinalysis. (A) Overall sum of all antibiotic days prescribed. (B) Days of treatment of different antibiotics by the appropriateness of urinalysis.

A total of 787 days of antibiotic treatment were prescribed across the patient cohort, 265 days (33.6%) were allocated to patients without a justified need for antibiotic treatment against a urinary pathogen or any other infectious cause (Figure 2a). Specifically, of the 203 total days on which third-generation cephalosporins were administered for UTI treatment in our cohort of 124 patients, 91 days (45%) were for patients without a justified need. Similarly, of the 51 days amoxicillin-clavulanic acid was prescribed for UTI indications in the full cohort, 21 days (41%) were for patients without an obvious indication (Figure 2b).

## Discussion

In this study, we reviewed 124 cases where urine cultures were obtained, and antibiotics were prescribed for UTIs and discovered that 31% of these cases were unnecessarily treated due to ASB.

UTIs are clinically diagnosed based on patient history.^5,17,18^ However, accurately diagnosing UTIs becomes challenging among patients who are unable to provide a clear history, as conditions like falls and altered mental status may mask an underlying infection.^19,20^ In such scenarios, UTIs often become a differential diagnosis, despite the high prevalence of abnormal urinalysis and positive urine cultures in these populations, which do not necessarily correlate with symptomatic UTIs.^21,22^

Analysis of the ASB cases in our cohort revealed that urinary infections were considered, and urinary cultures were often obtained based on nonspecific indicators such as cloudy or foul-smelling urine or during investigations of syncope or falls without direct symptoms indicating a UTI. In some instances, urinary tests were obtained, and antibiotics were prescribed in the setting of elevated white blood cell counts alone, without other obvious signs of infection.

The widespread treatment of ASB has led to the development of decision-support tools aimed at reducing unnecessary antibiotic treatment. However, the optimal point during the patient care process to implement these measures remains unclear and varies across practices. Many of these tools are based on reflex testing from urinalysis to culture, allowing for urine culture only if the urinalysis is abnormal.^16,23^ Despite these measures, a considerable number of patients with ASB continue to exhibit abnormal urinalysis results, suggesting that these tools may not always be adequate.^24–26^

Some interventions propose disregarding abnormal urinalysis and urine cultures altogether, especially when the initial decision to perform urinalysis is made under incomplete information, typically in high-pressure settings like EDs. In such approaches, educational efforts like the Canadian “Symptom-free pee, let it be” campaign inform staff about ASB and clear indications for treatment, even in patients who can provide only limited clinical history.^27^

Ideally, the decision-support tool should be used before obtaining a urine culture to reduce unnecessary workload and costs. However, real-life circumstances during patient care may necessitate using the tool only before initiating antibiotics, with the primary focus on reducing unnecessary antibiotic treatment. These considerations should be carefully considered when implementing this tool.

In our study, we found a high proportion of cases that would not warrant UTI screening using the 3-step clinical decision tool, 31% of the patients underwent unnecessary urine culture and received unnecessary antibiotic treatment. We did however have 1 case where urine cultures initially seemed unwarranted due to a clear diagnosis of pneumonia; however, upon retrospective examination, the presence of Enterobacteriaceae in both blood and urine suggested a concurrent UTI. The occurrence of UTI as a complication in patients diagnosed as having pneumonia, particularly prevalent among elderly individuals from nursing homes, has been previously documented.^28^ Thus, considering a urinary culture and providing care with antibiotics that cover urinary tract pathogens, in addition to treating the primary source of infection even in the face of an apparent pneumonia diagnosis, might be justified in selective cases.

This study has some limitations that warrant consideration. As a retrospective analysis, it depends on the completeness and accuracy of electronic health records, which might not capture the full extent of patient histories, especially in high-demand healthcare environments. Moreover, clinical guidelines recommend against obtaining a urine culture and treatment of a UTI when symptoms can be explained by other diagnoses, but complex clinical realities often involve patients with multiple concurrent issues, where an infectious process might trigger other medical events. Another limitation is the overlap between the 3-stage tool and the criteria used to assess the necessity of antibiotics when reviewing the cases. Although the 3-stage tool was assessed based on the information available in the EMR at the time of obtaining a urine culture and starting antibiotic treatment, the review assessed the full course of hospitalization. This overlap might have impaired the validity of the sensitivity and specificity calculations, which should be further examined in prospective studies.

In conclusion, our findings reveal a significant prevalence of ASB among patients treated with antibiotics for UTIs. We believe that implementing a straightforward 3-question model before obtaining a urine culture and antibiotic prescription could significantly diminish unnecessary antibiotic treatments. By doing this, we can potentially reduce associated harms with antibiotic exposure and, subsequently, their costs and the development of resistance. In addition, in the setting of acute hospitals, additional infectious work-up and treatment could prolong hospital length of stay. We advocate for further studies to validate the prospective implementation of this 3-question CDST to guide and enhance diagnostic and therapeutic antibiotic stewardship in the management of UTIs.
